# Clinical and lung function outcomes in a cohort of children with severe asthma

**DOI:** 10.1186/s12890-020-1101-6

**Published:** 2020-03-18

**Authors:** Patricia de Gouveia Belinelo, Aleisha Nielsen, Bernadette Goddard, Lauren Platt, Carla Rebeca Da Silva Sena, Paul D. Robinson, Bruce Whitehead, Jodi Hilton, Tanya Gulliver, Laurence Roddick, Kasey Pearce, Vanessa E. Murphy, Peter G. Gibson, Adam Collison, Joerg Mattes

**Affiliations:** 1 Priority Research Centre GrowUpWell, Hunter Medical Research Institute, University of Newcastle, Lookout Road, New Lambton, 2305 Australia; 2 Department of Respiratory Medicine, The Children’s Hospital at Westmead, Sydney, Australia; 3 Paediatric Respiratory & Sleep Medicine Department, John Hunter Children’s Hospital, Newcastle, Australia; 4 Priority Research Centre Healthy Lungs, Hunter Medical Research Institute, University of Newcastle, Newcastle, Australia; 5 Respiratory & Sleep Medicine Department, John Hunter Hospital, Newcastle, Australia

**Keywords:** Severe asthma, Lung function, Multidisciplinary clinic, Childhood asthma

## Abstract

**Background:**

Uncontrolled severe asthma in children is burdensome and challenging to manage. This study aims to describe outcomes in children with uncontrolled severe asthma managed in a nurse-led severe asthma clinic (SAC).

**Methods:**

This retrospective analysis uses data collected from children referred by a paediatric respiratory specialist to a nurse-led SAC for uncontrolled severe asthma between 2014 and 2019. The pre-clinical assessments included a home visit to assess modifiable factors that could be addressed to improve control. A comprehensive lung function analysis was conducted at each visit. Interventions were personalised and included biologic agents. Statistical analysis was performed using nonparametric, two-tailed Mann-Whitney U-test, the parametric Student’s t-test, or analysis of variance (ANOVA) as appropriate.

**Results:**

Twenty-three children with a median age of 12 years were seen once, and 16 were followed up. Compared to a non-asthmatic (NA) and asthmatic (A) age-matched cohort, children with severe asthma (SA) had a lower FEV1, and FVC% predicted before and after bronchodilator inhalation, and a higher mean Lung Clearance Index [LCI] (10.5 [SA] versus 7.3 [NA] versus 7.6 [A], *p* = 0.003). Almost 80% of children with SA had an abnormal LCI, and 48% had a reduced FEV1% at the first SAC visit. Asthma control and FEV1% predicted significantly improved at a follow-up visit, while LCI remained abnormal in the majority of children (83%).

**Conclusion:**

Over time, many children with severe asthma showed improved clinical outcomes and lung function while lung ventilation inhomogeneities persisted. Future appropriately controlled studies are required to determine if a nurse-led multidisciplinary SAC is associated with better outcomes.

## Background

Patients with severe asthma (SA) pose a significant challenge to healthcare professionals as it is a complex clinical problem with multiple contributing factors [1]. Uncontrolled SA in children encompasses those who are difficult to treat or have comorbidities, as well as those who are truly refractory to intervention [2, 3]. Difficult to treat asthma may occur as a result of poor medication adherence or inhaler technique, adverse environmental conditions (e.g., smoke, allergens, weather), and the child’s and family’s (lack of) knowledge of and attitudes towards asthma [4–6]. Comorbidities adversely affecting asthma control include obesity, allergic rhinitis, psychosocial factors, and obstructive sleep apnoea [7]. SA has a great impact on the quality of life (QOL) of children [8]. Management of uncontrolled SA may require a personalised treatment approach that involves a multidisciplinary team and is informed by a comprehensive set of clinical assessments. However, data on clinical outcomes employing this model of care in children with uncontrolled severe asthma are sparse.

This report, therefore, aims to retrospectively describe our “real world” experiences and outcomes managing children with severe asthma in the nurse-led severe asthma clinic (SAC) at the John Hunter Children’s Hospital, Newcastle, Australia.

## Methods

### Study design and participants

Data were collected retrospectively from March 2014 to March 2019 in the context of a clinical audit (Research ethics approval number AU201708–04). Patients were referred to the multidisciplinary SAC and seen on the basis of the clinical need. Referrals to the SAC were only accepted when made by a Paediatric Respiratory Specialist. Children were required to fulfil the European Respiratory Society (ERS) criteria for uncontrolled SA at the time of referral. Exceptions to those criteria included referrals of children who were not treated in accordance with GINA step 4 due to, for instance, prior side effects or lack of benefit with GINA step 4 treatment. No child was treated with biological agents before the first SAC visit because a comprehensive evaluation in the SAC was a prerequisite for the approval of prescribing biological agents in our department.

### Nurse-led home visit

Prior to the first appointment in the SAC, a home visit was conducted to identify modifiable factors that could be addressed in order to improve the child’s asthma control. The asthma clinical nurse consultant conducted an extensive assessment which included past medical history, inhaler check (technique and appropriateness of inhalers), adherence check, medication access, knowledge and understanding of asthma, indoor and outdoor allergen exposures, tobacco smoke exposure, allergic rhinitis, gastroesophageal reflux, child/family asthma management and the child’s perspective on their home and school life. Paediatric asthma quality of life questionnaire (PAQLQ) [9], Asthma Control Test (ACT) [10, 11], Asthma Control Questionnaire (ACQ-5) [12], Paediatric Index of Emotional Distress (PI-ED) [13], and Hospital Anxiety and Depression Scale (HADS) [14] were completed by the parent and child or nurse where appropriate.

SAC appointments (initial and follow-up) were attended by the asthma clinical nurse consultant, (a) paediatric respiratory specialist(s), and a general and adolescent paediatrician along with the parent(s)/carer(s) and the child. Future clinical management was determined after consideration of all available assessments by the multidisciplinary team in consultation with the family at the time of the visit.

### Control cohort

For some comparisons, data from the ongoing prospective birth cohort study Growing into Asthma (GIA) [15] were used. These children were born to asthmatic mothers who participated in the Management of Asthma in Pregnancy (MAP) study [16]. Children from the GIA cohort were followed-up at 10 years of age and stratified into children with and without doctor diagnosed asthma.

### Pulmonary function testing

For the SAC patients, lung function testing was performed as clinically indicated and determined by the Paediatric Respiratory specialist in attendance at each appointment. Before each measurement, weight, length, and body mass index (BMI) were recorded.

For both cohorts, all lung function testing was performed according to ATS/ERS recommendations [17, 18]. All measurements were carried out by qualified clinical respiratory scientists.

### Spirometry

Spirometry was performed using the Medisoft Whole Body Plethysmograph System in SAC children (Medisoft, Sorinnes, Belgium) distributed by Ascencia. Melbourne, Australia. Three forced vital capacity (FVC) manoeuvres were performed, and the best value of FVC and FEV1 was recorded. This technique was repeated pre and post inhalation of 4 doses of a bronchodilator (BD, Salbutamol 0.1 mg per dose) via a Breath a Tech Spacer (Medical Developments International, Victoria, Australia).

### Nitrogen multiple breath washout (N_2_ MBW)

N_2_ MBW testing was performed using a commercially available device (Exhalyzer® D, Eco Medics AG, Duernten, Switzerland; Spiroware version 3.1), which measures N_2_ concentration indirectly using simultaneous assessment of O_2_ and CO_2_ concentrations. The test was performed in a seated position whilst watching a movie to encourage stable tidal breathing. A facemask interface was used for preschool children whilst a mouthpiece and nose clip was used in older children. A minimum of two (ideally three) technically acceptable trials were collected. Technically acceptable trials were determined using published quality control criteria [19], based on consensus guideline recommendations [20]. A coefficient of variance (CoV) of within 10% for three trials and 5% for two trials was seen as reassuring of good data quality. Functional residual capacity (FRC) and the Lung Clearance Index (LCI) were reported as the mean of technically acceptable trials. The reference data within the software was used to define normality [21].

### Skin prick test

Skin prick testing (SPT) was conducted using the list of allergens (commonly requested *Dermatophagoides pteronyssinus, Dermatophagoides farinae, grass and tree pollen mix, cat and dog dander, and* Aspergillus *fumigatus)* requested by the Respiratory Specialist. A positive result was defined as 3 mm or greater than the negative saline control, determined by averaging maximal perpendicular wheal diameters 15 min after applying the lancet. The positive control was histamine base, 6 mg/ml (Stallergènes®, Antony, France), and had to be 3 mm or greater for a valid test. Patients were asked to withhold any antihistamines for 2 days prior to the test. GIA participants had their SPT standardised,and *Dermatophagoides pteronyssinus, Dermatophagoides farinae*, *cat, dog, Aspergillus fumigatus, grass and tree pollen mix, and the standard food allergens (whole peanut extract, egg yolk, and egg white)* were used.

### Blood tests

Blood tests such as total serum Immunoglobulin E (IgE), specific serum IgE (RAST), and blood eosinophils were requested if never done previously. Results were then checked and considered positive if higher than the normal range for each specific test.

### Statistical analysis

Categorical measures were summarised using counts and percentages, while continuous measures were summarised using means, median, standard deviations, and range. Statistical significance was determined using a nonparametric, two-tailed Mann-Whitney U-test or the parametric Student’s t-test as appropriate. For the analysis of matched pair parameters, the paired t-test was performed.

Analysis of variance (ANOVA) was used to determine whether there were any statistically significant differences between the means of two or more independent (unrelated) groups, and when overall significance was discovered Post hoc testing was performed using a multiple comparison test.

## Results

### Subject characteristics

Twenty-five children were managed in the SAC between 2014 and 2019. Of those, two patients under the age of 4 years were excluded in this analysis due to the uncertainty of asthma diagnosis in this age. In regards to family history, 30% (*n* = 7) had a history of maternal asthma, 13% (*n* = 3) had a history of paternal asthma, 9% (*n* = 2) had a history of both maternal and paternal asthma, while 39% (*n* = 9) had no history of parental asthma. For two children, no information about parents’ health status was available. The baseline characteristics were compared to 20 children from the GIA birth cohort study, of whom 7 children had mild to moderate asthma, and 13 were not diagnosed with asthma (Table 1).

**Table 1:** Characteristics of SAC children and asthmatic and non-asthmatic GIA children

	Severe Asthma Clinic children (*n* = 23)	GIA children with asthma (*n* = 7)	GIA children without asthma (*n* = 13)	*p* value
	Mean/median (SD/min-max)	Mean/median (SD/min-max)	Mean/median (SD/min-max)	
Age (years)	**11.4**/12 (2.7/4–16)	**10.1**/10 (0.3/10–11)	**9.8**/10 (0.37/9–10)	0.070
Weight (kg)	**47.2**/46.3 (19.2/20.3–110)	**42.7**/39.9 (10.6/28.5–60.3)	**35.5**/32.6 (8.6/27.4–58.5)	0.107
Height (cm)	**147.7**/151.6 (26.03/105.6–168.5)	**145.5**/146. 5(7.2/136.5–156)	**138.2**/138 (8.6/125–152)	0.123
BMI^a^	**20.9**/19.8 (5.5/14–39.9)	**19.9**/18.9 (3.5/16.2–26.3)	**18.4**/17.8 (3.3/13.9–26.5)	0.323
ACT^b^	**14.2**/12 (5.4/8–25)	**22.4**/24 (3.1/16–25)	–	*0.0006*
	**n (%)**	**n (%)**	**n (%)**	
Number (%) of ACT^b^ score ≤ 19	**17**(74)	**1**(14)	–	*0.005*
Male gender	**12**(52)	**5**(71)	**5**(38)	0.582

^a^*BMI* body mass index, ^b^*ACT* Asthma Control Test score

While the median age was 10 to 12 years in all cohorts, the range was greater in the SAC cohort as expected, with the youngest child being 4 years of age and the oldest 16 years of age. The median ACT was 15 in the SAC cohort with some SAC patients (*n* = 6 out of 25) having had satisfactory asthma control (score > 19) in the past 4 weeks before their first SAC visit.

Further clinical characteristics of the SAC cohort are shown in Table 2. Median onset of asthma symptoms was at 1.5 years of age, and half of all SAC children were admitted to hospital for asthma in the past 12 months with a median number of three hospitalisations in the previous year. Two of those patients also had admissions to the Paediatric Intensive Care Unit (PICU) in the previous year.

**Table 2:** Disease burden and results of investigations in Severe Asthma Clinic patients

Severe Asthma Clinic children (*n* = 23)	
Hospital Admissions in the previous year	**10** (43)^c^
Number of Hospital admissions in the previous year	**3**/3(0.9/1–4)^d^
Number of children with Positive Skin Prick Test	**19** (83)^c^
Number of children with serum Specific IgE positive	**18** (78)^c^
● Aspergillus	**4** (22) ^c^
● D pteronys	**15** (65) ^c^
● Australia treemix	**4** (22) ^c^
● Cat epithelia	**7** (30) ^c^
● Dog dander	**9** (50) ^c^
● Weed mix	**4** (22) ^c^
● Grass mix	**7** (30) ^c^
● Mould mix	**4** (22) ^c^
Blood eosinophils levels^a^	**909**/800 (66/0–2900) ^d^
Serum Immunoglobulin E (IU/mL) ^a^	**1257**/1210 (1125/165–4565)^d^
Age at symptoms onset in years^a^	**3.7**/1.5 (3.9/0.5–12) ^d^
Age at visit date in years	**11.4**/12 (2.7/4–16) ^d^
ICS^b^ (mcg/day)	**1851**/2000 (642/640–4000) ^d^
Number of children with ACT score ≤ 19	**17** (74) ^c^
ACT score	**14.2**/12 (5.4/8–25) ^d^
Quality of Life Questionnaire score ^a^	**5.2**/5.3 (1.1/3.1–7) ^d^
ACQ-5^a^	**1.8**/1.8 (1/0–3.8)^d^
PI-ED	**9**(38)^c^
● Anxiety ● Depression	**5**/3 (6.6/0–20) **0.6**/0 (1.3/0–3)
Number of children under treatment with Azithromycin	**7** (30) ^c^
Nasal steroids	**16 (70)**^c^
Bronchoscopy	**9** (39^c^)
Bronchoalveolar Lavage Fluid (BALF)^a^	**7** (30) ^c^
● % Macrophage	**86.4**/88 (12.6/68–99.5) ^d^
● % Eosinophil	**2.8**/1.3 (7.5/0.5–21) ^d^
● % Lymphocyte	**7.9**/7.5 (8.8/0.5–26) ^d^
● % Neutrophil	**4.7**/1 (8.0/0.5–19) ^d^

^a^BALF analysis n = 7; serum IgE n = 23; age at symptoms onset in years n = 15; blood eosinophils levels n = 21; Quality of Life questionnaire n = 13; ACQ-5 n = 14

^b^*ICS* Inhaled corticosteroid;^c^Values presented as n (% of total)

^d^Values presented as Mean/median (SD/min-max)

The PAQLQ has 23 questions in 3 domains (symptoms, activity limitation, and emotional function), and children were asked to think about the previous week and respond to each of the 23 questions on a 7-point scale (7 = not bothered at all; 1 = extremely bothered). The median PAQLQ score was five and ranged from 3 to 7 (Table 2).

The median daily dose of inhaled corticosteroids was 2000mcg beclomethasone equivalents ranging between 640 and 4000mcg. Most children were atopic (*n* = 20 out of 23 positive in SPT or RAST) and had blood eosinophilia (*n* = 17 out of 23 with > 300 cells/mcl). A subpopulation of SAC children underwent bronchoscopy (*n* = 9) subsequent to the first visit with the main purpose to characterise their type of airways inflammation and to assess for pathogen growth (*n* = 8) and/or to exclude airway malformations (*n* = 1) (Table 2).

### Lung function at first SAC visit

Lung function data at baseline are summarised in Table 3. FEV1% predicted, and FEV1/FVC% were significantly lower in SAC children both before and after bronchodilator inhalation as compared to children with doctor-diagnosed asthma and children without asthma even if BD response tended to be largest in the SAC children. In those children where N_2_ MBW was performed (*n* = 14, 61%), LCI was significantly higher in SAC children versus those with or without an asthma diagnosis (Table 3). The proportion of the cohort with an LCI outside of the normal range was higher than FEV1% (79% versus 52%, respectively, Table 3)*.* Correlation between LCI and FEV1% (rs = − 0.699, *p* = 0.005, *n* = 14) showed to be significant, while correlation between LCI and residual volume to total lung capacity percentage ratio (RV/TLC%; “trapped air”) was not significant (r = 0.402; *p* = 0.173; *n* = 13).

**Table 3:** Pulmonary function test at first SAC visit compared to GIA

	Severe Asthma Clinic children	GIA children with asthma	GIA children without asthma	
	*n* = 23 Mean/median (SD/min-max)	*n* = 7 Mean/median (SD/min-max)	***n*** = 13 Mean/median (SD/min-max)	p value
FVC litres pre BD	**2.6**/2.6 (0.7/1.3–4.0)	**2.4**/2.4 (0.5/1.9–3.4)	**2.3**/2.4 (0.4/1.6–3.1)	0.546
FVC % pre BD	**97.7**/98 (14.9/64–127)	**93.5**/94.5 (11.6/75.2–111)^$^	**108.5**/109.2 (10.5/84.1–124)^$^	*0.019*
FEV1 litres pre BD	**1.8**/1.9 (0.6/1.1–3.3)	**2.1**/2.0 (0.3/1.7–2.6)	**1.9**/2.1 (0.3/1.2–2.6)	0.300
FEV1% pre BD	**78.4**/81 (17.7/40–112) *	**94.9**/95 (5.8/86–103)	**102.7/**104.8 (12.13/72–121)*	*0.0001*
Abnormal FEV1% pre BD^#^	**11**(48)	**0**	**1**(8)	*0.007*
FEV1/FVC litres pre BD	**70.3**/69.4 (9.5/47.7–88.2)^!^	**88.0**/84.1 (7.9/78.1–98.4)^!^	**121.3**/119.4 (8.1/110.3–135.2)^!^	*< 0.0001*
FVC litres post BD	**2.8**/2.7 (0.8/1.3–4.1)	**2.4**/2.5 (0.5/1.9–3.3)	**2.4**/2.4 (0.4/1.7–3.2)	0.189
FVC % post BD	**102.3**/104 (13.22/75–124)	**95.9**/98.1 (9.8/82–109)^$^	**109.9**/110.2 (8.7/90–122.3)^$^	*0.011*
FEV1 litres post BD	**2.0**/2.1 (0.6/1.1–3.5)	**2.2**/2.2 (0.3/1.8–2.7)	**2.1**/2.2 (0.4/1.3–2.8)	0.458
FEV1% post BD	**85.7**/88 (16.7/47–117)*	**99.2**/99.8 (4.8/92–106)	**109.6**/112 (12.2/78.1–129.2)*	*< 0.0001*
FEV1/FVC litres post BD	**73.7**/73.7 (9.6/55.7–92.3)^!^	**89.3**/90.2 (5.5/79.9–96.1)^!^	**87.1**/89.6 (5.1/75.9–94.4)^!^	*< 0.0001*
BDR %	**11**/7 [11.3/(−2)-45]	**4**/6 [4.3/(−1.2)-10]	**7**/7 (3.7/0–13)	0.187
BDR ≥12%^#^	**6**(26)	**0**	**1**(8)	0.158
TLC%	**115**/111 (21/88–184)	**–**	**–**	–
TLC litres	**4**/4 (1/3–6)	**–**	**–**	–
RV/TLC %	**157**/152 (42/92–254)	**–**	**–**	*–*
Abnormal RV/TLC%^#^	**13**(57)	**–**	**–**	*–*
	**n = 14**	**n = 7**	***n*** **= 11**	
LCI	**10.5**/10.7 (2.3/7.1–13.9)^!^	**7.6**/7.4 (1.2/6.3–9.9)^!^	**7.3**/7.3 (1.0/5.4–9.1)^!^	*0.003*
Abnormal LCI (%)^@^	**11**(79)	**1**(14)	**3**(27)	*0.002*
FRC/body weight (mL/kg)	**47/**46 (15/23–76)	**36**/35 (10/20–50)	**44**/42 (14/21–72)	0.211
FeNO (ppb)**	**31.7**/15 (31/6–90)	**31**/20 (25/12–74)	**24**/21 (19/7–51)	0.861
Abnormal FeNO^#^	**8**(38)	**2**(40)^#^	**2**(40)^#^	0.934

^@^LCI in a health cohort is ≤ 8 [20, 22, 23]

^#^Values presented as n (% of total); ** FeNO n = 21;Only 5 participants from each GIA group had valid FeNO results.*Significant between SAC and GIA without asthma; ^$^Significant between both GIA groups

^!^Significant between SAC and GIA with asthma, and also between SAC and GIA without asthma

### SAC follow-up visit

Nine SAC children were not followed-up; reasons included that they were unable to attend the follow-up (*n* = 1) and have not yet attended their appointment (*n* = 8). The follow-up assessment was performed after an average of 26 months from the first visit (median 32, range 4 to 56 months). In the 16 SAC children who had a follow-up SAC visit, a significant improvement in FEV1% predicted, FEV1/FVC %, and ACT score were observed while the reduction in LCI (11.0 to 9.5, *p* = 0.237) was not significant in the 6 subjects with N_2_ MBW at two occasions (Table 4).

**Table 4:** Pulmonary function test at first visit and follow-up of SAC children who attended both visits

Severe Asthma	First Visit	Follow-up	
	***n*** **= 16** **Mean/median (SD/min-max)**	***n*** **= 16** **Mean/median (SD/min-max)**	**p value**
FVC litres pre BD	**2.5**/2.6 (0.6/1.6–3.7)	**3.7**/3.4 (1.3/1.4–6.7)	*0.0002*
FVC % pre BD	**92.2**/95.5 (13.1/64–109)	**100.6**/101 (10.2/81/114)	0.053
FEV1 litres pre BD	**1.7**/1.7 (0.4/1.1–2.5)	**2.7**/2.1 (0.9/1.2–4.9)	*< 0.0001*
FEV1% pre BD	**69.8**/71.5 (14.4/40–91)	**84.1**/85 (10.9/59–107)	*0.0009*
Abnormal FEV1% pre BD	**10**(63)	**5**(31)	0.077
FEV1/FVC litres pre BD	**66.9**/67.3 (8.7/47.7–88.4)	**75.3**/74.0 (8.4/64.7–95.3)	*0.002*
FVC litres post BD	**2.6**/2.7 (0.6/1.6–3.9)	**3.8**/3.5 (1.3/1.5–6.8)	*0.0002*
FVC % post BD	**97.7**/102 (12.1/75–117)	**103.5**/105 (9.6/84–116)	0.136
FEV1 litres post BD	**1.8**/1.8 (0.4/1.2–2.7)	**2.9**/2.9 (1.0/1.3–5.6)	*0.0003*
FEV1% post BD	**78.5**/71.2 (13.7/47–98)	**89.9**/92 (13.2/66–118)	*0.006*
FEV1/FVC litres post BD	**71.2**/72.3 (9.3/55.7–92.9)	**76.5**/76.2 (10.6/54–98.3)	0.073
BDR %	**13**/8 (13/3–45)	**8**/6 [7.7/(−4)-23]	0.224
BDR ≥ 12%^a^	**5**(31)	**6**(38)	0.710
ACT score^b^	**14**/12 (5/8–25)	**18**/18 (5/12–25)	*0.009*
Percentage of ACT score ≤ 19^a^	**12**(75)	**9**(56)	0.264
TLC%	**117**/113 (24/88–184)	**105**/104 (13/86–140)	0.094
TLC litres	**4**/4 (1/3–6)	**11**/5 (24/2–101)	0.262
RV/TLC %	**171**/181 (40/112–254)	**131**/130 (32/77–199)	*< 0.0001*
Abnormal RV/TLC%	**12**(75)	**5**(31)	*0.013*
	**n = 6** **Mean/median (SD/min-max)**	**n = 6** **Mean/median (SD/min-max)**	
LCI	**11.0**/10.7 (1.7/8.6–13.6)	**9.5**/8.5 (2/7.2–12.8)	0.237
Abnormal LCI (%)^a^	**6**(100)	**5**(83)	0.297
FRC/body weight (mL/kg)	**51.9**/52.9 (10.5/38–62.3)	**58.4**/59.5 (17.9/34–79.9)	0.104
FeNO (ppb)^b^	**30**/19 (26/6–87)	**36**/31 (33/4–100)	0.390
Abnormal FeNO^a^	**5**(42)^b^	**6**(50)^b^	0.682

^a^Values presented as n (% of total)

^b^FeNO results n = 12; ACT score n = 15

Seven SAC children were commenced on biologics (*n* = 4 omalizumab; *n* = 3 benralizumab) before the follow-up SAC visit, while nine children received intervention without commencement of biologics (Table 5). Non-biologic treatment interventions were very diverse. They included nurse-led educational, behavioural and environmental interventions, and changes in medication (e.g., inhaler device, treatment of allergic rhinitis, azithromycin as needed [24]).

**Table 5:** Pulmonary function test first and follow-up for SAC patients stratified for intervention

	Intervention including biological treatment	Intervention including biological treatment		Intervention excluding biological treatment	Intervention excluding biological treatment	
	n = 7 Mean/median (SD/min-max)	n = 7 Mean/median (SD/min-max)	p value	n = 9 Mean/median (SD/min-max)	n = 9 Mean/median (SD/min-max)	p value
FVC litres pre BD	**2.6**/2.1 (0.6/1.8–3.7)	**3.9**/3.5 (1.5/2.5–6.7)	*0.0255*	**2.5**/2.6 (0.6/1.6–3.5)	**3.5**/3.4 (1.2/1.4–5.3)	*0.004*
FVC % pre BD	**88.1**/86 (16.5/64–109)	**106.1**/110 (8.1/95–114)	*0.0318*	**95.3**/96 (9.5/81–108)	**96.2**/93 (9.7/81–110)	0.809
FEV1 litres pre BD	**1.7**/1.8 (0.5/1.1–2.5)	**2.8**/2.4 (1.2/1.7–4.9)	*0.0258*	**1.7**/1.7 (0.4/1.1–2.4)	**2.7**/2.8 (0.7/1.2–3.4)	*0.0005*
FEV1% pre BD	**68.0**/71 (19.3/40–91)	**82.4**/78 (15.5/59–107)	0.0787	**71.2**/72 (10.1/57–84)	**85.4**/87 (6.1/73/92)	*0.004*
Abnormal FEV1% pre BD	**5**(71)	**4**(57)	0.5770	**6**(67)	**1**(11)	*0.016*
FEV1/FVC litres pre BD	**65.7**/66.8 (8.8/47.6–73.4)	**71.8**/69.4 (4.8/67.6–80)	0.1240	**67.9**/67.7 (9.1/57.6–88.2)	**78.0**/77.6 (9.8/64.7–95.3)	*0.012*
FVC litres post BD	**2.8**/2.8 (0.6/2.1–3.9)	**3.9**/3.6 (1.5/2.6–6.8)	*0.0295*	**2.6**/2.7 (0.6/1.6–3.7)	**3.7**/3.4 (1.2/1.5–5.5)	*0.004*
FVC % post BD	**94**/91 (14.4/75–113)	**108.3**/109 (6.5/97/116)	*0.0282*	**100.7**/102 (9.7/85–117)	**99.8**/102 (10.2/84–114)	0.837
FEV1 litres post BD	**1.9**/1.9 (0.5/1.2–2.7)	**2.9**/2.7 (1.4/1.6–5.6)	0.0631	**1.8**/1.7 (0.4/1.2–2.5)	**2.8**/2.9 (0.7/1.3–4.1)	*0.0007*
FEV1% post BD	**75.1**/79 (17.2/47–94)	**90**/85 (18.4/66–118)	0.0952	**81.1**/77 (10.7/89–98)	**89.8**/93 (8.5/73–98)	*0.009*
FEV1/FVC litres post BD	**68.7**/71.4 (8.1/55.7–79.6)	**72.2**/74.3 (9.9/54–82.6)	0.5572	**72.5**/72.9 (10.1/58.9–92.3)	**79.1**/75.7 (10.6/65.1–98.3)	*0.037*
BDR %	**13**/6 (16/3–45)	**13**/12 (7/3–23)	> 0.9999	**9**/9 [9/(−4)-29]	**5**/4 [6/(−4)-18]	0.243
TLC%	**117**/110 (31/88–184)	**104**/100 (1/92–101)	0.3830	**117**/114 (18/90–147)	**106**/105 (15/86–140)	0.096
TLC litres	**4**/4 (1/3–6)	**19**/5 (36/3–101)	0.3308	**4**/4 (1/2–6)	**5**/5 (2/2–7)	*0.009*
RV/TLC %	**167**/166 (53/112–254)	**131**/135 (42/77–199)	0.0579	**175**/181 (28/125–222)	**132**/127 (25/98–184)	*< 0.0001*
Abnormal RV/TLC %	**4**(57)	**3**(43)	0.5930	**8**(89)	**3**(33)	*0.016*
ACT score^a^	**14**/14 (6/8–21)	**19**/20 (6/12–24)	0.2486	**15**/15 (5/8–25)	**19**/19 (5/13–25)	*0.022*
Percentage of ACT score ≤ 19 ^a^	**4**(67)	**3**(50)	0.6963	**7**(78)	**5**(56)	0.317
	***n*** **= 5**	**n = 5**		**n = 2**	**n = 2**	
LCI	**11.1**/10.9 (2/8.6–13.6)	**9.6**/8.3 (2.4/7.2–12.8)	0.3435	**12.4**/12.4 (2.7/10.5–14.3)	**8.5**/8.5 (0.3/8.3–8.7)	–
Abnormal LCI (%)	**5**(100)	**4**(80)	0.2918	–	–	–
FRC/body weight (mL/kg)	**50**/47 (11/38–62)	**56**/48 (18/34–79)	0.2347	**49**/49 (10/41–58)	**60**/60 (10/50–70)	–
FeNO (ppb)^a^	**32**/25 (29/8–87)	**42**/49 (29/6–76)	0.4576	**27**/16 (24/6–60)	**31**/14 (37/4–100)	0.728
Abnormal FeNO^a^	**3**(50)	**4**(67)	0.5582	**2**(33)	**2**(33)	–

^a^ACT score n = 6; FeNO n = 6

Improvements in lung function were significant for FVC % predicted before and after BD in the cohort of children treated with biologics (Table 5). In contrast, improvements in lung function were significant for FEV1% predicted and FEV1/FVC % but not FVC % predicted before and after BD in the cohort of children receiving intervention that did not include biologics (Table 5).

## Discussion

This retrospective analysis describes clinical audit data exploring outcomes in children with uncontrolled SA managed in a nurse-led SAC, which included lung function parameters and lung ventilation inhomogeneities. Our SAC was largely implemented in accordance with the Royal Brompton Hospital London SAC model [25] with changes made to best suit local resources and clinical needs. For instance, we accepted referrals of children younger than 6 years and tested for true steroid-resistant asthma using oral steroids only in exceptional circumstances. Interestingly, prior to their first SAC visit, all children had been managed by paediatric respiratory specialists who were all part of the multidisciplinary SAC team and case discussions. Despite this, it seems likely that some children had difficult-to-treat rather than therapy-refractory asthma. Those difficult to treat asthmatic children were identified by a nurse-led assessment, including a home visit and evidence-based tools, and have responded to nurse-led interventions such as improvement of inhaler technique, compliance, and medication availability at home and in school, and control of environmental symptom triggers. A proportion of them was not further followed-up in the SAC. It could be hypothesised that the nurse-led assessment was an effective means to identify treatment difficulties more effectively. Indeed the “simple” steps of checking for adherence to therapy concurrent with asthma education can markedly improve asthma control and promote a better quality of life for some patients [8, 26, 27]. Our analysis was not appropriately controlled to provide high-level evidence for a beneficial effect of a nurse-led SAC on outcomes. Specifically, we did not have a cohort of children with SA not managed in a nurse-led SAC in our Department. This would be critical as Ross and co-workers have recently shown in a cohort of children with uncontrolled SA that half of those children no longer had severe asthma after 3 years [28]. Therefore future studies elucidating the efficacy of interventions in children with SA require appropriate control groups.

Independent of what intervention was pursued after the first SAC visit, the majority of children referred to our SAC demonstrated a significant disease burden evidenced by asthma onset in early childhood, a history of numerous past hospital admissions for asthma attacks, and clinically significant reductions in ACT, ACQ and PAOLQ scores. 61% of children with SA had a parental history of asthma. Increased asthma severity in this cohort was supported by reduced lung function parameters, including a higher prevalence of lung ventilation inhomogeneities when compared to a cohort of children with doctor-diagnosed asthma and children without asthma. Children with SA also had a high prevalence of “trapped air” when measuring lung volumes. Previously abnormal spirometry has not been observed as highly discriminatory for different severities of asthma in children [25, 29] and our results support this by showing that an abnormal LCI appears more discriminatory (e.g., Table 3: 79% abnormal LCI versus 48% abnormal FEV1% in SAC children). The novel finding of highly abnormal LCI values in children with SA is consistent with recent specialised imaging studies that have described the presence of significant ventilation inhomogeneities with hyperpolarized gas MRI imaging, with the degree of abnormality associated with asthma severity and due to subsegmental narrowing or complete obstruction of small airways [30, 31].

Improvements were observed in ACT and lung function parameters before and after BD inhalation, and a reduction in “trapped air” was apparent. Stratifying the analysis of the SA cohort by treatment with biologic agents resulted in small case numbers per group. It is possible that this may have resulted in a lack of power to show that either group had greater improvements over the other. However, following a protocol with a standard set of investigations may have allowed for a more personalised management approach and could explain the significant improvements in asthma control and lung function of most children independent of what that intervention was.

Our results suggest that LCI may have improved, although not significantly, at the follow-up SAC visit. However, for the majority of patients, LCI remained in the abnormal range (83%) even after a personalised asthma intervention. Abnormal LCI measurements have been demonstrated in children with normal spirometry [32]. More studies are required to elucidate the precise mechanisms underpinning the occurrence of lung ventilation inhomogeneities in SA and the clinical relevance of its persistence. Possibly, an optimal treatment modality for some children with severe therapy-refractory asthma has yet to be identified, and monitoring changes in LCI may be a sensitive marker for the persistence of small airway disease. A number of biologics have been trialled, mostly in adults, to treat severe asthma with a type (T)2-high inflammatory response, including mAb directed against IgE (omalizumab), IL-5 (mepolizumab), IL-5 receptor alpha (benralizumab), IL-13 (lebrikizumab and tralokinumab) and the IL-4 receptor alpha chain (dupilumab) [33, 34]. In children with severe asthma, however, increased submucosal expression of IL-33, an epithelial-derived alarmin, and IL-33 positive non-residential cells are found in the airway wall. IL-33 levels were correlated with reticular basement membrane (RBM) thickness, which is considered the result of subepithelial fibrosis [35]. Investigating the efficacy of a combination of dupilumab and SAR440340, an antibody directed against IL-33, may shed light on the role of IL-33 in severe childhood asthma [36]. Subsegmental narrowing or complete obstruction of small airways observed in SA may adversely affect the deposition of inhalant anti-inflammatory and bronchodilating drugs. It could then be speculated that the severity of lung ventilation inhomogeneities may be associated with asthma that is more refractory to inhalant therapies.

## Conclusion

In conclusion, we audited our SAC data and observed significant improvements in asthma control and lung function over time. We observed clinical improvements independent of the modality of the specific intervention when managed in a nurse-led multidisciplinary paediatric SAC. Children with severe asthma demonstrated lung ventilation inhomogeneities that persisted in most despite significant improvements in other clinical and lung function outcomes. Further studies are required to assess the efficacy of a nurse-led SAC and the value of LCI in monitoring airway disease in severe asthma.

## Data Availability

The datasets used and/or analysed during the current study are available from the corresponding author on reasonable request.

## Abbreviations

ACQ: Asthma Control Questionnaire
ACT: Asthma Control Test
ANOVA: Analysis of Variance
ATS: American Thoracic Society
BD: Bronchodilator
BMI: Body Mass Index
ERS: European Respiratory Society
FEV1: Forced expiratory volume in one second
FRC: functional residual capacity
FVC: Forced Vital Capacity
GIA: Growing into Asthma
GINA: Global Initiative for Asthma
HADS: Hospital Anxiety and Depression Scale
IgE: Immunoglobulin E
LCI: Lung Clearance Index
mAb: Monoclonal antibodies
MAP: Management of Asthma in Pregnancy
MRI: Magnetic resonance imaging
PAQLQ: Paediatric asthma quality of life questionnaire
PI-ED: Paediatric Index of Emotional Distress
QOL: quality of life
SA: severe asthma
SAC: severe asthma clinic
